# 2240: High-throughput phenotyping and the increased risk of OSA in Rosacia patients

**DOI:** 10.1017/cts.2017.62

**Published:** 2018-05-10

**Authors:** Peter Elkin, Sarah Mullin, Sanjay Sethi, Shyamashree Sinha, Animesh Sinha

**Affiliations:** University at Buffalo, State University of New York, Buffalo, NY, USA

## Abstract

OBJECTIVES/SPECIFIC AIMS: To create a new semantically correct high-throughput phenotyping (HTP) platform. To demonstrate the utility of the HTP platform for observational research and can allow clinical investigators to perform studies in 5 minutes. To demonstrate the improved accuracy of observational research using this platform when compared with traditional observational research methods. To demonstrate that patients who have Roseacea are at increased risk of having obstructive sleep apnea (OSA). METHODS/STUDY POPULATION: This population is a set of 212,343 patients in the outpatient setting cared for in the Buffalo area over a 6-year period. All records for these patients were included in the study. Structured data was imported into an OMOP (OHDSI) database and all of the notes and reports were parsed by our HTP system which produces SNOMED CT codes. Each code is designated as a positive, negative or uncertain assertion and compositional expressions are automatically generated. We store the codified data 750,000,000 codes in Berkley DB, a NOSQL database, and we keep the compositional graphs in both Neo4J and in GraphDB (a triple store). Labs are coded in LOINC and drugs using RxNorm. We have developed a Web interface in .Net named BMI Search, which allows real-time query by subject matter experts. We analyzed the accuracy of structured Versus unstructured data by identifiying NVAF cases with ICD9 codes and then looked for any additional cases based on the SNOMED CT encodings of the clinical record. This was validated by 2 clinical human review of a set of 300 randomly selected cases. Separately we ran a study to determine the relative risk of OSA with and without Rosacea using the data set described above. We compared the rates using a Pearson χ^2^ test. RESULTS/ANTICIPATED RESULTS: We are able to parse 7,000,000 records in an hour and a half on 1 node with 4 CPUs. This yielded 750,000,000 SNOMED CT codes. The HTP data set yielded 1849 cases using ICD9 codes and another 873 using the HTP-NLU data, leading to a final data set of 2722 cases from our population of 212,343 patients. In total, 580 patients had Rosacea;5443 patients had OSA without Rosacea and 51 patients had OSA with Rosacea. Patients with Rosaca had an 8.8% risk of OSA whereas patients without Rosacia only had a 2.6% risk of OSA. This was highly statistically significant with a *p*<0.0001 (Pearson χ^2^ test). The number needed to test was only 12. DISCUSSION/SIGNIFICANCE OF IMPACT: HTP can change how we do observational research and can lead to more accurate and more prolific investigation. This rapid turn around is part of what is necessary for both precision medicine and to create a learning health system. Patients with Rosacea are at increased risk of and should be screened for OSA.

